# Indirect effects of higher mean air temperature related to climate change on major life-history traits in a pulsed-resource consumer

**DOI:** 10.1038/s41598-026-37071-3

**Published:** 2026-01-23

**Authors:** Lukas Hochleitner, Shane Morris, Maximilian Bastl, Thomas Ruf, Claudia Bieber

**Affiliations:** 1 Research Institute of Wildlife Ecology, Department of Interdisciplinary Life Sciences, University of Veterinary Medicine, Vienna, Austria; 2 Department of Oto-Rhino-Laryngology, Medical University of Vienna, Vienna, Austria

**Keywords:** Temperature change, Resource pulse, Glis glis, Life-history, Hibernator, Seed consumer, Ecology, Climate-change ecology

## Abstract

Climate change is directly and indirectly affecting species. The degree of these effect types differs by species and context, with indirect effects likely to be stronger for consumers of pulsed resources. Here, we investigated how higher mean air temperature related to climate change affects masting, and in parallel, how this change affects life-history traits in edible dormice (*Glis glis*). We analysed 17 years of capture-recapture data from 2,530 individuals. We collected air temperature, and, as a measure of seed production, pollen data from European beech (*Fagus sylvatica*). Our results show that increasing mean air temperature was associated with a shift in beech pollen production, leading to a biannual mast cycle in recent years, with alteration of years with very high and very low seed availability. The changed cycle in mast events resulted in a significant reduction in overall yearling survival in dormice, while overall adult survival remained stable. In parallel, both age classes significantly increased their litter size in this timeframe. Furthermore, survival probabilities in the two age classes also differed depending on the beech mast status (mast, mast-failure). We show that the observed dramatic changes in seed production had complex effects on life-history traits in a pulsed resource consumer.

**Supplementary Information:**

The online version contains supplementary material available at 10.1038/s41598-026-37071-3.

## Introduction

Climate change has far-reaching direct and indirect effects on plant and animal species globally^1,2^. The future intensification of these effects will prompt major environmental changes, resulting in population declines and species extinctions^3,4^, as well as in species range expansions and increasing population density^5,6^. Several studies have observed the indirect effects of climate change on life-history traits, particularly those linked to survival and reproduction, in different animal populations^7–9^. So far, few studies have explored these indirect effects on life-history traits in species in pulsed resource systems^10,11^, where climate change seems to have an even stronger disruptive effect^12,13^.

Climate change has an even greater influence in pulsed resource systems, since most pulsed resources are triggered by specific weather cues, e.g., temperature^14,15^. Varying resource availability occurs in all ecosystems and creates responses in successive trophic levels of ecological communities and can be major drivers of population dynamics^16,17^. Pulsed resource systems are characterized by their extreme episodes of unpredictable and infrequent resource availability^18^. One such system is the masting of seed trees in temperate forests, where in so called mast years, a certain proportion of masting trees within an area produce large amounts of seeds^18^. Vetter et al.^19^ proposed a classification, which defines mast years as years in which more than 30% of the trees in an area produce seeds. These mast years were further divided into intermediate and full mast years, depending on whether less (intermediate mast) or more than 85% (full mast) of the trees are producing seeds. Full mast years reflect a high synchrony of all beech trees in a certain area^19^. Two strategies are assumed to drive this pulsed resource system: *i*) in wind-pollinated tree species, this strategy guarantees a high degree of fructification, *ii*) seed consumers are overwhelmed by this high seed availability and cannot adapt to this pattern, leading to a high seed survival^20,21^. However, some mammals, like the American red squirrel (*Tamiasciurus hudsonicus*), eastern chipmunk (*Tamias striatus*), and edible dormouse (*Glis glis*) are highly adapted to plants’ pulsed seed reproduction. All three species rely in so called mast years (high seed production as a large scale phenomenon) on these seeds as a primary food resource and increase their investment into reproduction in these years^22–24^.

Animals may adapt their life-history strategies to changed patterns of pulsed resource production, which can be explained by re-allocation of the available resources between major life-history traits, like survival and reproduction^25,26^. In general, higher reproductive investments are traded against somatic maintenance^27^ and/or survival probability^28^. However, there are adaptations and certain lifestyle conditions, which allow even small mammals to increase their chance of survival, and thus future reproduction^29^. For example, the ability to fly or arboreality are lifestyles which substantially affect trade-offs in allocation, due to an increased probability to escape predation^30^. An extreme example of this survival strategy in mammals is hibernation. Hibernators enter torpid states, such as hibernation in winter or estivation in summer (with torpor bouts lasting more than 24 h), in response to unfavourable environmental conditions (e.g., food or water shortages, or low temperatures)^31^. Inactive in concealed underground burrows, increases survival, not only through reducing energy expenditure but also due to predator avoidance^29,32^. The duration of hibernation is very flexible and can be extended up to ~ 11 months under certain conditions^33^.

In this study, we examined the life-history traits in edible dormice (hereafter dormice), small arboreal and omnivorous hibernators, which are highly adapted to the seed pulses of European beech (*Fagus sylvatica*, hereafter beech)^34,35^. The beech, a mast-seeding tree, primarily relies on the mean summer temperature in years prior to masting as a cue^36^. Furthermore, in beech, airborne pollen abundance is a good predictor of autumn seed production^37^. In years with high beech seed availability, dormouse survival rates are lower due to high reproductive costs in both sexes^32^. In contrast, in years with low beech seed availability, entire dormouse populations skip reproduction by remaining in a state of reproductive quiescence due to the lack of available beech seeds^35^. An increased survival probability during these years through prolonged phases of estivation and hibernation^33^ results in dormice having a longer lifespan, up to 14 years, than expected for their size^38^. These prolonged phases of estivation are indicated by lower recapture probabilities in years with low beech seed availability, shown in several populations across Europe^32^. Indeed, considering the aspect that an animal is alive but cannot be recaptured is essential in estimating correct survival probabilities in hibernating species^39,40^.

For our study we used capture-mark-recapture data from 2,530 dormice captured in 135 nest-boxes over 17 years in the Vienna Woods, Austria. We investigated whether higher mean air temperature related to climate change affected dormice indirectly by an altered beech masting cycle associated with higher air temperatures. Investigating indirect effects of rising temperatures on a species´ life-history traits is a difficult approach, especially in field studies. Thus, we analysed in a first step the effect of mean air temperature on beech pollen production. To examine the effects of an altered beech masting cycle due to higher mean air temperature on life-history traits in dormice, we split the investigation period into two distinct temperature phases - a cooler Period 1 (2006–2013) and a warmer Period 2 (2014–2022). We analysed whether food availability, using annual pollen data as a proxy, was higher in the warmer phase, thus, increasing the proportion of reproductively active females and/or litter size compared to the cooler phase. We hypothesized, based on life-history theory, a reduced survival for dormice because of increased reproductive investment due to higher food availability in Period 2. Based on this, we investigated whether the survival probabilities of the two age classes, yearlings and adults, show different patterns in the two distinct temperature periods. In this context, we hypothesized that adults might have a potentially lower survival due to higher reproductive investments compared to yearlings, since adult female dormice have been shown to produce larger litters than yearlings^40^. Larger litters are associated, in general, with higher maternal reproductive costs^28^, which can lower the survival of the mother^41^.

## Results

### Effects of air temperature on Beech

**Fig. 1:**
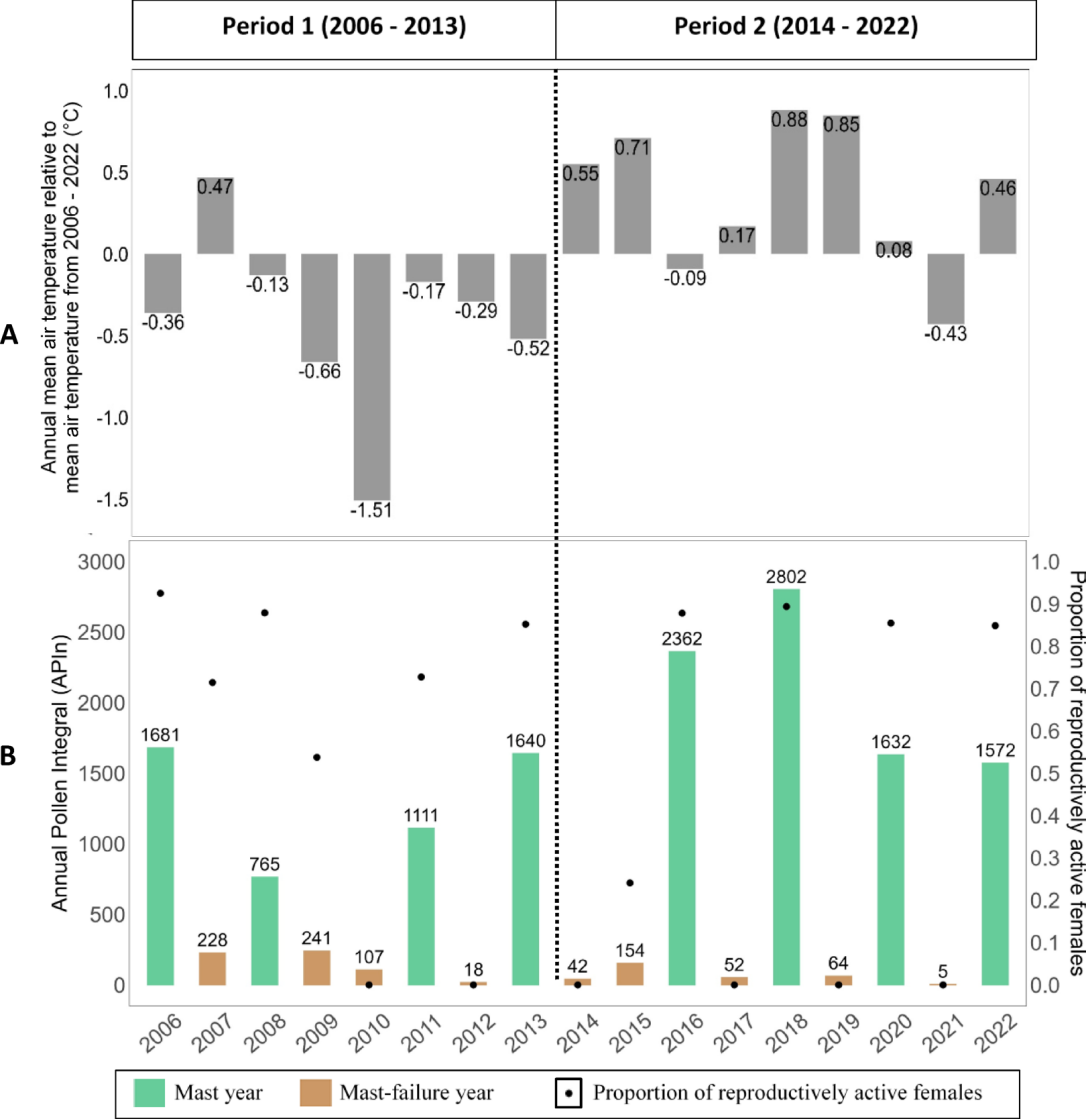
Abiotic and biotic environmental characteristics in relation to reproductive output of edible dormice (*Glis glis*) during Period 1 (2006–2013) and Period 2 (2014–2022). (**A**) Annual mean air temperature (in °C) from 2006 to 2022 relative to the mean air temperature of the study period (= 8.53 °C). (**B**) Annual Pollen Integral (APIn) of European beech (*Fagus sylvatica*) in relation to the proportion of reproductively active female dormice per year from 2006 to 2022. In the years 2010, 2012, 2014, 2017, 2019 and 2021 no reproductively active females were captured. Since airborne pollen abundance is a good predictor of beech seed production, APIn was used as a proxy for seed availability in each respective year. Mast years were characterized by > 593 pollen grains/m^3^/year (see Methods).

The largest annual mean air temperature difference of 0.75 °C was observed by splitting the dataset into Period 1 (2006–2013) and Period 2 (2014–2022; see Methods). Average air temperature was 8.14 °C in Period 1 and 8.89 °C in Period 2 (t-test: t-value_1,15_ = 3.06, *p* = 0.0079; annual data see Fig. 1A). The average beech Annual Pollen Integral (APIn) was higher in Period 2 (965 pollen grains/m^3^/year) than in Period 1 (724 pollen grains/m^3^/year), with the difference becoming even more pronounced comparing only mast years across the periods (Period 2: 2092 pollen grains/m^3^/year, Period 1: 1299 pollen grains/m^3^/year; Fig. 1B). The beech APIn increased with increasing mean summer temperature in the year before the mast event (F-value_1,15_ = 7.37, *p* = 0.0160). Inter-annual variation in APIn was greater in Period 2 (coefficient of variation (CV) = 1.17) than in Period 1 (CV = 0.09; *p* = 0.0014; Fig. 1B). In Period 2, a maximum of 2802 pollen grains/m^3^/year occurred (the highest measured APIn during the last 46 years, Supplementary Fig. 1), while the maximum in Period 1 was much lower (1,681 pollen grains/m^3^/year).

### Survival-recapture-probability models

The best survival-recapture-probability model was ɸ (age class*sex + age class*period + mast*period) P (age class*period*mast + sex) (Table 1). Since the second-best, third-best, and fourth-best models had a ΔQAICc of less than 2 (Table 1), these models were also considered. All these models included an interaction between “mast” and “period”. Additionally, in the second-best model, these two variables interacted with “age class” as well.

**Table 1:** Model selection table for local annual survival and recapture probability.

Model-rank	Survival parameters	Recapture parameters	K	QAICc	ΔQAICc	Model-likelihood
1	ɸ (age class * sex + age class * period + mast * period)	*P* (age class * mast * period + sex)	19	5156.42	0.00	1.00
2	ɸ (age class * mast * period + age class * sex)	*P* (age class * mast * period)	20	5156.84	0.42	0.81
3	ɸ (age class * sex + age class * period + mast * period)	*P* (age class * mast * period + sex * mast)	20	5157.51	1.09	0.58
4	ɸ (age class * sex + age class * period + mast * period)	*P* (age class * mast * period + sex * age class)	20	5158.41	1.99	0.37
	ɸ (~ 1)	*P* (~ 1)	2	5445.08	288.66	0.00

Models are ranked according to their QAICc. Models include additive effects (“+”) and interactions (“*”). K = number of estimated parameters; QAICc = quasi-likelihood corrected AICc (c-hat = 1.2528075); ΔQAICc = difference between model QAICc and QAICc of best model; Model likelihood = relative strength of evidence for a model within the set of models.

### Local survival probability and litter size

**Fig. 2:**
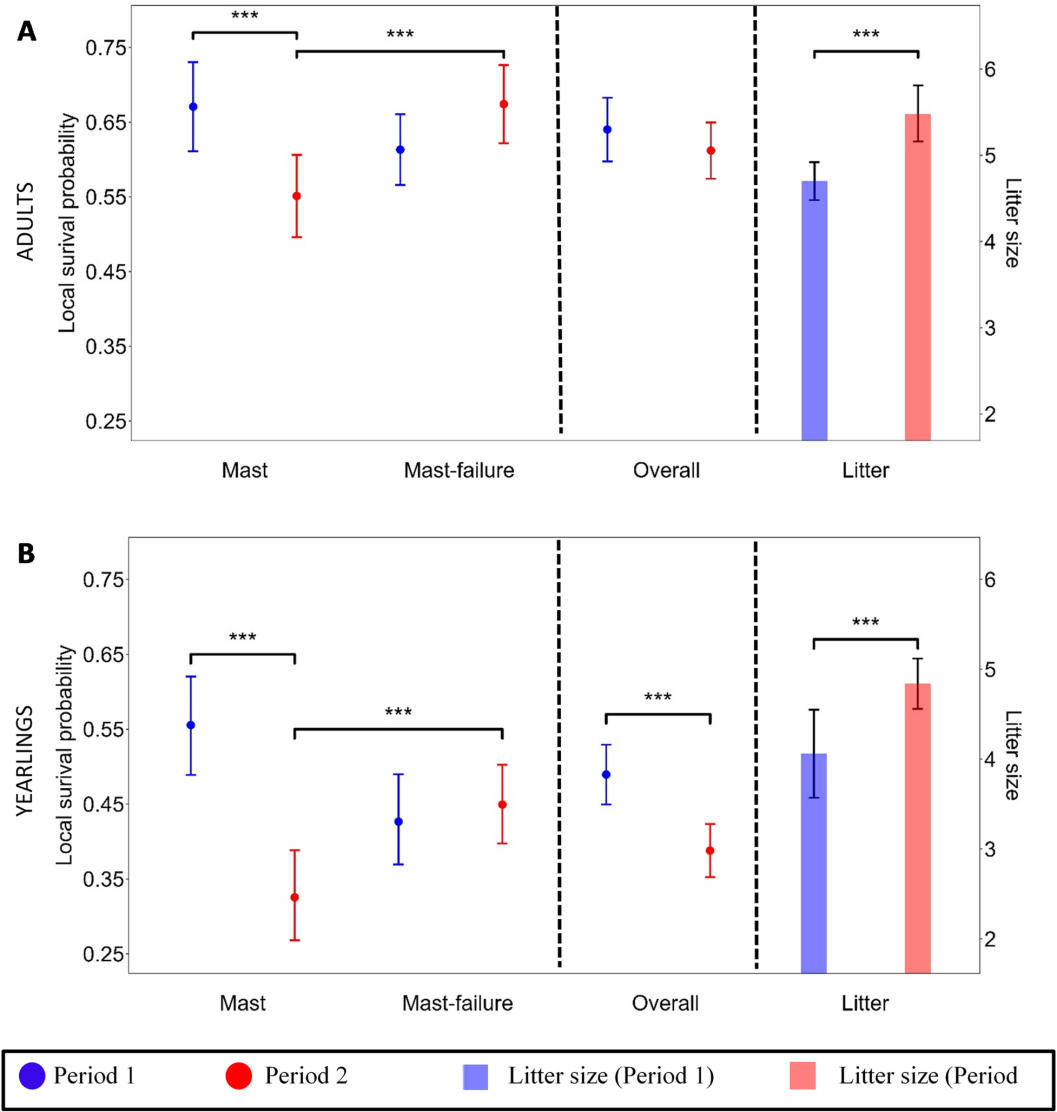
Local annual survival probability (mean estimate with 95% confidence intervals) of dormice (*n* = 2,530) and mean litter size (*n* = 242) during Period 1 (2006–2013) and Period 2 (2014–2022). (**A**) Local annual survival probability during mast and mast-failure years (second-best capture-recapture model), overall local annual survival probability (best capture-recapture model), and litter size (mean ± SE of linear model) of adults. (**B**) Local annual survival probability during mast and mast-failure years (second-best capture-recapture model), overall local annual survival probability (best capture-recapture model), and litter size (mean ± SE of linear model) of yearlings. Stars and brackets indicate statistically significant differences, but no absolute p-value can be determined when using MARK.

While yearlings survived significantly better in Period 1 (0.49, CI = 0.45–0.53 ) than in Period 2 (0.39, CI = 0.35–0.42, Fig. 2B, best model), adults’ survival probabilities did not significantly differ between the two periods (Period 1: 0.64, CI = 0.60–0.68 ; Period 2: 0.61, CI = 0.57–0.65 ; Fig. 2A, best model). In parallel, dormice had significantly larger litters in Period 2 (adults = 5.48 ± 0.32, yearlings = 4.84 ± 0.28) compared to Period 1 (adults = 4.70 ± 0.22, yearlings = 4.07 ± 0.49; F-value_1,239_ = 7.12, *p* = 0.0082; Fig. 2A and B). Splitting the data into mast and mast-failure years, it becomes evident that yearlings and adults survived mast years significantly better in Period 1 (yearlings: 0.56, CI = 0.49–0.62 ; adults: 0.67, CI = 0.61–0.73 ) than in Period 2 (yearlings: 0.33, CI = 0.27–0.39; adults: 0.55, CI = 0.50–0.60 ; Fig. 2A and B, second-best model). There was no significant difference of survival in mast-failure years between Period 1 (yearlings: 0.43, CI = 0.37–0.49 ; adults: 0.61, CI = 0.57 -0.66) and Period 2 (yearlings: 0.45, CI = 0.40-0.0.50; adults: 0.68, CI = 0.62–0.73; Fig. 2A,B). During Period 2, yearlings and adults survived significantly better in mast-failure years (yearlings: 0.45, CI = 0.40–0.50; adults: 0.68, CI = 0.62–0.73) compared to mast years (yearlings: 0.33, CI = 0.27–0.39; adults: 0.55, CI = 0.50–0.60 ; Fig. 2A,B, second-best model). In Period 1, there was no significant difference in survival between mast-failure years (yearlings: 0.43, CI = 0.37–0.49 ; adults: 0.61, CI = 0.57–0.66) and mast years (yearlings: 0.56, CI = 0.49–0.62; adults: 0.67, CI = 0.61–0.73 ; Fig. 2A,B). Furthermore, in adults, females (0.69, CI = 0.66–0.73) survived significantly better than males (0.56, CI = 0.52–0.60, Supplementary Fig. 2, best model).

### Recapture probability

Adults’ recapture probabilities were significantly higher in mast years (Period 1: 0.63, CI = 0.55–0.71; Period 2: 0.75, CI = 0.69–0.82) compared to mast-failure years (Period 1: 0.45, CI = 0.39–0.52; Period 2: 0.35, CI = 0.30–0.40; Supplementary Fig. 3). In contrast, yearlings’ recapture probabilities did not differ significantly between mast (Period 1: 0.69, CI = 0.58–0.79; Period 2: 0.61, CI = 0.50–0.71 ) and mast-failure years (Period 1: 0.48, CI = 0.37–0.59 ; Period 2: 0.64, CI = 0.52–0.74 ; Supplementary Fig. 3).

### Reproductive activity of females and litter size

**Fig. 3:**
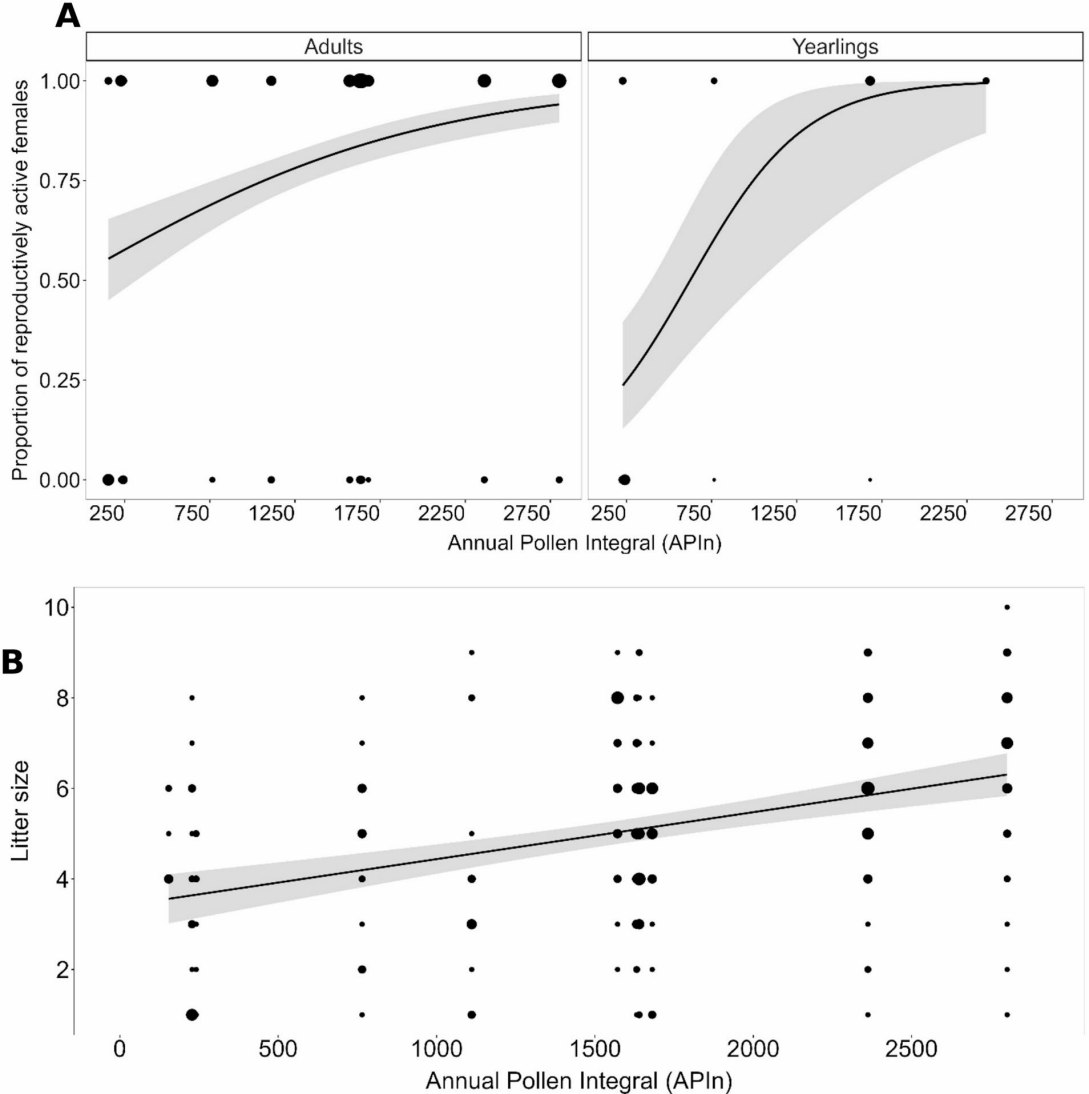
Reproductive patterns in female edible dormice. (**A**) Proportion of reproductively active female adults (*n* = 380) and yearlings (*n* = 58) increased with a rising Annual Pollen Integral (APIn) of beech of the current year (z-value_1,435_ = 2.40, χ² = 5.74, *p* = 0.0166). A general linear mixed model (GLMM) with a binominal error distribution and logit link function was built. (**B**) Litter size (*n* = 242) of dormice increased with beech’s APIn of the current year (t-value_1,239_ = 3.09, χ² = 9.53, *p* = 0.0020). A linear model (LM) was built to explain variation in litter size. The plots show the model’s effect size. The grey bands represent the 95% confidence intervals, and the size of the black dots represents the number of observations.

We found a strong correlation between mast events and reproductively active female dormice (Pearson’s correlation: rho = 0.87, t-value_1,15_ = 6.92, *p* < 0.0001; Fig. 1B). Furthermore, the proportion of reproductively active females (*n* = 438) was affected by an interaction of beech APIn of the current year with age class (z-value_1,435_ = 2.40, χ² = 5.74, *p* = 0.0166). The proportion of reproductively active adult females steadily increased, from about 55% of adults reproducing at low APIns to nearly all adults reproducing at high APIns (Fig. 3A). In contrast, only about 25% of yearlings were reproductively active at low APIns, but the proportion of reproductively active yearlings increased strongly with higher APIns (Fig. 3A). Litter size (*n* = 242 litters) was positively affected by beech APIn of the current year (t-value_1,239_ = 3.09, χ² = 9.53, *p* = 0.0020; Fig. 3B) and was larger in Period 2 (Period 1: 4.61 ± 0.24, Period 2: 5.48 ± 0.36, t-value_1,239_ = 2.40, χ² = 5.77, *p* = 0.0163; Fig. 2A and B).

### Body mass of dormice

**Fig. 4:**
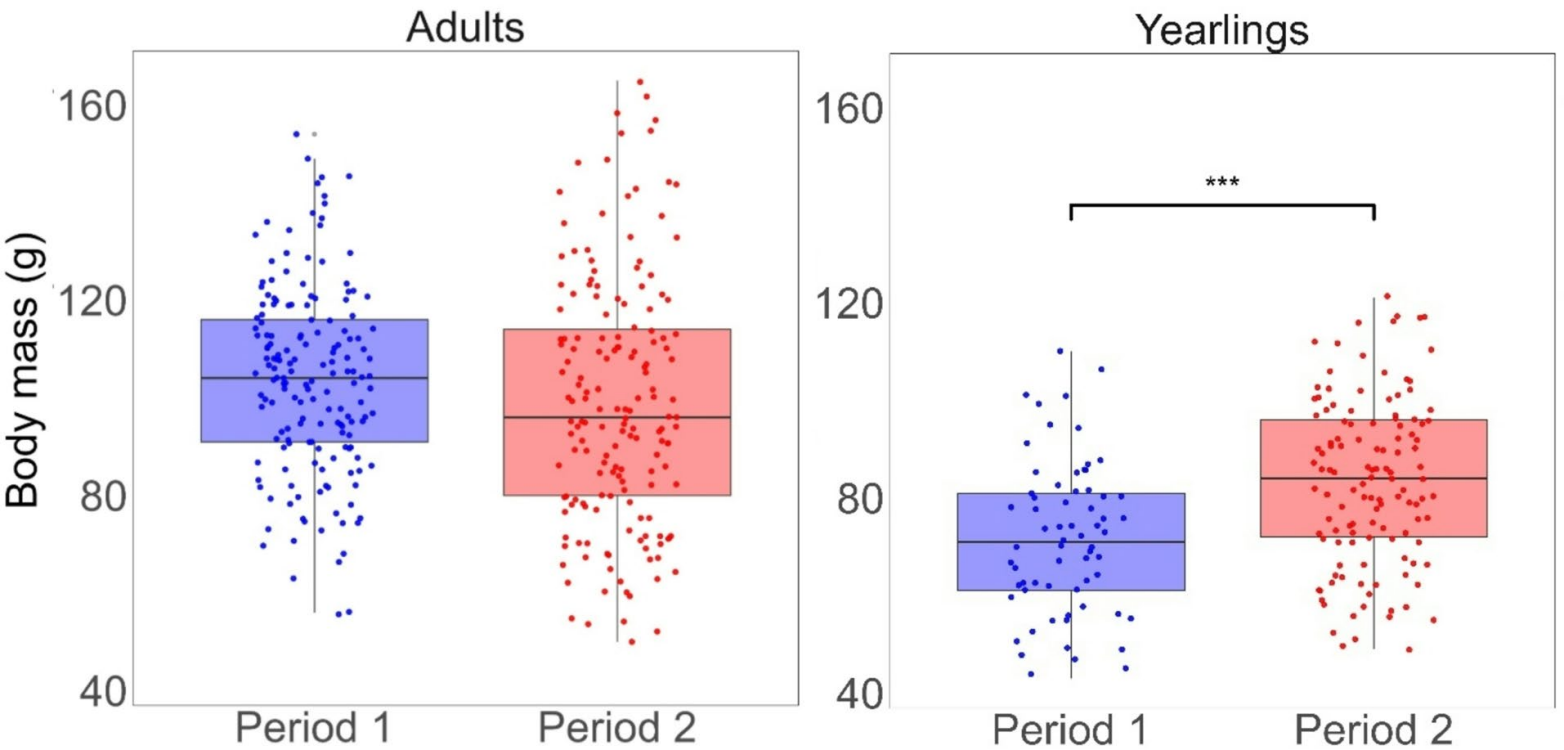
Dormouse body mass (in g) at the start of the active season across periods. Body mass of adults (*n* = 331) and yearlings (*n* = 190) at the start of the active seasons in Periods 1 and 2, captured within the first three weeks after the first capture of the active season (= first capture after hibernation). A Type III ANOVA was performed to compare body mass of yearlings and adults. Afterwards, the function “glht” in the R-package “multcomp” was used to assess specific group differences. Stars and brackets indicate statistically significant differences (*p* < 0.0001).

At the start of the active season, yearlings had significantly lower body mass than adults in both periods (Period 1: estimate_1,517_ = 32.20, t-value = 10.82, *p* < 0.0001; Period 2: estimate_1,517_ = 15.51, t-value = 6.33, *p* < 0.0001), but they showed significantly higher body mass in Period 2 compared to Period 1 (estimate_1,517_ = 11.81, t-value = 3.78, *p* = 0.0007; Fig. 4). Adult body mass did not significantly differ between the two periods (estimate_1,517_ = 4.88, t-value = 2.16, *p* = 0.1255; Fig. 4).

## Discussion

Here, we found an effect of higher mean air temperature related to climate change on seed masting, which then had a complex effect on life-history trade-offs between survival and reproduction in a pulsed-resource consumer. The beech seed production pattern changed within our study period, associated with increasing air temperature. Our data showed the highest APIns paralleled by high seed availability in two-year intervals in Period 2 compared to lower APIns in Period 1 (Fig. 1B) and generally in the past (Supplementary Fig. 1). Modelling survival indicated strong associations of period (a colder Period 1 compared to a warmer, more recent Period 2), mast status, age class, and sex with dormouse survival. The best model indicated differences in survival probabilities in the two age classes yearling and adult depending on the period, with the lowest survival in yearlings in Period 2. One of the best models (Model 2) included the three-way interaction between period, mast status, and age class, indicating that survival probabilities in the two age classes also differed depending on the beech mast status (mast, mast-failure). Furthermore, adult females had higher survival than adult males, likely due to higher reproductive costs in males than in females^35^.

Our findings of increased APIns associated with rising summer temperatures and greater inter-annual variation in pollen production in Period 2 align with results in other beech forests^12^. Compared to previous decades, highly productive mast years have become more frequent in beech in the UK, Germany, and Switzerland^42^. In Japan, higher summer temperatures in years before mast events have been shown to trigger higher future seed production^42^ (but see ^12^). In the past, mast seeding in beech trees at the study site appeared to be less strongly synchronized, resulting in several consecutive years with intermediate seed availability. In more recent year (as observed in Period 2) beech trees showed stronger synchrony with a high proportion of tree producing large seed crops in the same year, and that every second year. An increased synchrony might reflect an elevated resource stress or less cueing of favourable years, potentially caused by climate change^12^. In pulsed resource systems, effects of increasing temperature on pulsed-resource consumers are transmitted through changes in the mast cycle, as mast-seeding trees are affected by the rising temperatures due to climate change^10,42^. In comparison, in other types of environments, higher temperatures directly affect species, such as in yellow bellied marmots (*Marmota flaviventris*), which had earlier emergence dates after hibernation at higher burrow temperatures^43^. We did not find a similar effect of temperature on emergence in this study (see Supplementary Table 1). However, the indirect pathway - via temperature driven changes in the seed production pattern - can have significant consequences in species that depend on mast. For instance, in mast-consuming wild boars (*Sus scrofa*), an increased frequency of masting events positively impact population growth^44^.

To unravel the relationship between masting changes and seed consumer survival, it is important to understand what is happening during both mast and mast-failure years. During mast years, adult and yearling survival was lower in Period 2 than in Period 1 (Fig. 2). Larger litters in Period 2 (Fig. 2) likely resulted from increased food availability, indicated by higher APIns. Although lactation is energy-intensive for females^28^, more offspring can be produced if energy-rich food is more abundant^37^ (Fig. 3B). Unlike other seed consumers, e.g., the non-hibernating American red squirrel^22^, dormice did not invest in a second litter in response to higher seed availability due to the restricted active season in hibernators. Juveniles need to gain sufficient body fat reserves for their first hibernation^40^. Other species, like the Columbian ground squirrel (*Urocitellus columbianus*), use higher food availability to store energy by accumulating additional mass for the subsequent reproductive season^27^. However, higher reproductive investments are expected to increase foraging activity to meet additional energy demands, as seen, e.g., in rabbits (*Oryctolagus cuniculus*), increasing their time spent feeding with higher litter sizes^45^. Increased foraging activity is further associated with higher predation risk, which has been shown in general for rodents, like in northern Idaho ground squirrels (*Urocitellus brunneus*)^46^ or in Merriam’s kangaroo rats (*Dipodomys merriami*)^47^, and in particular for hibernators^48^. This association, coupled with energetic constraints from reproduction, may explain lower dormouse survival in mast years in Period 2.

In mast-failure years, adult and yearling survival did not differ significantly between the periods, but adults generally survived better than yearlings in both periods (Fig. 2). Higher adult survival can be explained by findings that individuals with higher body mass can prolong estivation during the active season in years with reproductive skipping, i.e., mast-failure years with very low beech seed availability^33^, as an efficient predation-avoidance strategy to increase survival^48^. In a subtropical mammal, the northern long-eared bats (*Nyctophilus bifax*), it has been shown that individuals with higher body condition employ torpor to avoid predation when they do not need to feed^49^. Indeed, in dormice, body mass strongly correlates with body fat^50^. Recapture probabilities in adults were lower in mast-failure years compared to mast years (Supplementary Fig. 3), indicating that adults retreated in years with both low beech seed availability and no reproduction into underground hibernacula during the active season^51^. Yearlings showed no differences in recapture probabilities between mast and mast-failure years (Supplementary Fig. 3). Indeed, yearlings were significantly lighter than adults (Fig. 4), which might be due to a smaller body size but also due to smaller fat reserves. It has been shown that animals with lower body mass stayed more active and did not estivate to the same extent^33^. In other hibernating species, direct negative effects of a warmer climate have resulted in increased activity and reduced time in prolonged torpor, lowering survival^52^. Since survival probabilities in mast-failure years did not differ significantly between the two periods in our study, it seems that time spent in prolonged torpor was unaffected. This again emphasizes that the impacts of higher mean air temperature on the studied dormouse population were mediated by the beech mast cycle .

Overall, adults maintained high survival in Period 2 despite increased reproductive investments. Hibernators can increase their energy intake and store more body fat through resistance to appetite-suppressing hormones and boosting lipid-storing pathways^53^. Thus, hibernators have unique physiological adaptations to use time-limited high resource availability to store energy for increased investments in both reproduction and survival^43^. Higher reproductive investments combined with an increased availability of beech seeds in mast years most likely explain the significant difference in survival between mast and mast-failure years in Period 2 (in contrast to the non-significant difference in Period 1; Fig. 2). Increased reproductive investment reduced the survival during mast years in Period 2 compared to Period 1 (significant difference, Fig. 2). However, the high seed availability in mast years in Period 2 may have enabled more females to acquire sufficient body mass after pup rearing and prior hibernation onset. Consequently, in the consecutive mast-failure year, more females may have emerged from hibernation with higher body mass and entered prolonged hibernation earlier, slightly increasing their survival during mast-failure years in Period 2 compared to Period 1. Such a significant difference between low- and high-reproductive years was previously found in a dormouse population in Germany between 1993 and 2001^40^. The Vienna Woods covers the foothills of the eastern Alps and therefore higher altitudes than the warm regions of southwest Germany (450–500 m a.s.l., approximately 1.5 °C higher average annual mean air temperature from 1991 to 2020). Thus, effects of higher mean air temperature on the mast cycle and, consequently, dormouse survival may have been delayed at our study site. However, the results justify the assumption that effects of the mast cycle on seed consumers are a large-scale phenomenon, with flexible life-history adaptations in this species, not a locally restricted genetic adaptation^32^.

Our results reveal that, unlike adults, yearlings had lower survival probabilities in Period 2 than in Period 1 (overall survival, Fig. 2). Thus, our study suggests that indirect effects of higher mean air temperature can differ between age classes. Yearlings must invest, additionally to reproduction and survival, in covering costs related to somatic growth^25,54^. Columbian ground squirrel yearlings have been shown to only invest in both somatic growth and reproduction once their daily energy intake increased, while adults with high body mass did not need additional energy to reproduce because they could use accumulated energy i.e. body mass^54^. In our study, yearlings were significantly lighter after hibernation in Period 1 compared to Period 2 as well as compared to adults (in both periods; Fig. 4). Thus, yearlings might invest additional energy rather into growth than survival (i.e., accumulating body fat to enter estivation earlier). In general, investments in growth benefit future torpor periods, as larger individuals can store more body fat^55,56^ and thus increase the time spent in torpor (i.e., increase survival)^33^. In mast years, yearlings also primarily invest in growth (smaller litter sizes than adults), but it seems that in Period 2 sufficient resources were available to increase reproductive investments without compromising somatic growth and/or storing body fat^39,57^. However, the increased reproductive investments in Period 2, may explain the lower survival in yearlings in Period 2 compared to Period 1. Whether this changing pattern in yearling survival will affect future population dynamics and longevity in this species, is not clear yet.

The shift to a two-year cycle of alternating years with very high and very low beech seed availability observed in Period 2 (see also^58^ will result in juveniles being born only every second year in our population. Consequently, in this two-year cycle yearlings will always face years with low beech seed availability, providing sufficient food to increase body mass but insufficient food resources to allow successful reproduction^33^. In contrast, in Period 1, yearlings had in multiple years (2006, 2007, 2008, 2009) the opportunity to reproduce. Thus, this above described two-year cycle will delay first reproduction to the second year of life - a long period to survive for small mammals not able to exhibit estivation as predation-avoidance-strategy. However, it is known that dormice do live in forests without seed-producing beech trees, e.g., at the edge of the distribution range, and thus, they can adjust their breeding performance to a changing environment and food availability^37^.

Our study focused on changes of survival and reproduction in a pulsed resource consumer, the edible dormouse. In this context, we can highlight the importance of the mast cycle on life-history strategies in pulsed resource consumers like dormice. The direct effects of higher mean air temperature on the beech as seed producer are connected to changes in the successive trophic level, namely the seed consumers. Admittedly, a limitation of our study is that it is observational and can not conclusively determine the causality between direct or indirect pathways of higher mean air temperature effects on life-history patterns. Further research, potentially involving experimental manipulations under captive conditions, would be needed to establish causality. An experimental setup could look like the following: testing the effect of different temperatures at the same level of food availability^59^, on the one hand, and testing different levels of food availability under the same temperature regime^60^, on the other hand. However, our findings strongly suggest that temperature change-induced changes in the mast cycle impact trade-offs between reproduction and survival in dormice. While other pulsed resource consumers are likely to face similar effects, our study also reveals that general conclusions are difficult to draw. In our study, investigating a hibernating seed consumer (adapted to pulsed resources) indicated that as many facets of a species lifestyle and life-history traits as possible should be considered for meaningful statements. In dormice, adults increased investment into reproduction in the warmer period, while yearlings, due to smaller body fat reserves for prolonged torpor and investment into growth, showed a reduced survival in the warmer period. This led to different survival probabilities among age classes and periods. Ongoing climate warming may further alter beech mast patterns, for instance, leading to smaller reproductive events as plants struggle to fill up their resource reserves sufficiently between more frequent mast events or even a potential breakdown of mast seeding^42,61^. It is entirely unclear what a possible breakdown in beech masting will cause in temperate forest ecosystems in the future. Our study, however, provides first insights into the complex system of seed producers and seed consumers under higher mean air temperature related to climate change.

## Methods

### Study site and data collection

We evaluated data from an ongoing capture-mark-recapture research program in the Vienna Woods near St. Corona (Lower Austria, 48°04’ N, 15°56’ E, WGS84) from 2006 to 2022. The study area (500–800 m a.s.l.) is covered by a deciduous mixed forest dominated by beech stands (for more details, see^33,62^. In total, 135 nest-boxes were installed in the study area, affixed 2–4 m high on trees along forest trails. Nest-boxes were checked during daytime, once a fortnight, throughout dormice’s active season (late April - mid October). Dormice hibernate in underground hibernacula^63^. At each capture event, captured individuals were weighed (to the nearest 1 g using a 300 g balance, PESOLA, Switzerland), and classified by age class, sex, and reproductive state. Newly captured individuals were marked with a subcutaneously injected transponder (TIERCHIP DASMANN©, 8.0 × 1.4 mm or BackHome BioTec©, 13.8 × 2.1 mm). Age classification into juveniles (before first hibernation), yearlings (after first hibernation), and adults (after second hibernation) was based on fur colour^24^. Yearlings can be sexually mature when they are not fully grown^24^. Females with visible nipples and captured together with juveniles in the same nest-box were classified as reproductively active. Females captured at least twice between week 31 and week 39 of the year (time of rearing young^39^ without visible nipples and juveniles in the same nest-box were categorized as non-reproductively active. A total of 438 females were used in the analysis of the proportion of reproductively active females. An additional 195 females were excluded, since these females were captured just once (without visible nipples and juveniles in the same nest-box) or were never captured between week 31 and week 39 in years with reproduction. We recorded 254 litters in total, of which 242 litters were used in the analysis. The other 12 litters of communal breeding (defined as more than one reproductively active female with visible nipples in a nest-box with juveniles) were excluded from the analysis due to unknown maternity. All captured dormice were promptly returned to their nest-box after measurements. This study was approved by the Ethics and Animal Welfare Committee of the University of Veterinary Medicine, Vienna, in accordance with the University’s guidelines for Good Scientific Practice and authorized by the Austrian Federal Ministry of Education, Science and Research (ref. BMBWF 68.205/0075-WF/V/3b/2015) in accordance with current legislation.

### Air temperature

Mean daily air temperature was calculated from hourly recorded air temperature data measured in our study area (one single spatial point, 48°03’49’’ N, 15°55’28’’ E, WGS84). Due to measurement errors, we lacked air temperature for 768 days out of a total of 6209 days. To address this gap, these missing days’ daily mean air temperatures were estimated. Our estimations were based on a strong correlation (Pearson’s correlation: rho = 0.97, *p* < 0.0001; Supplementary Fig. 4) between the air temperature in our study area and that recorded at the nearest weather station in Berndorf (coordinates: 47°56’ N, 16°06’ E, approximately 19.5 km from the study area). Daily mean air temperature for missing days was calculated with the formula “-1.463075+ (mean daily air temperature in Berndorf*0.950787)”. The weather station in Berndorf provided continuous data on daily mean air temperature for the entire duration of our study (data availability: https://data.hub.geosphere.at/dataset/klima-v1-1d).

### Soil temperature

Mean daily soil temperature was measured directly in our study area (one single spatial point, 48°03’49’’ N, 15°55’28’’ E, WGS84) at approximately 60 cm underground in hourly intervals, but only in some years. The depth of 60 cm was selected, since another study on this population showed that dormice hibernate at least 60 cm below the surface^33^. In total, we lacked soil temperature for 1419 days out of a total of 6209 days. Mean daily soil temperature of the missing days was estimated based on a strong correlation (Spearman correlation: rho = 0.89, *p* < 0.0001) between the measured mean daily soil temperature and the previous day’s mean daily air temperature (see “Air temperature”). The estimated values were calculated with the formula “3.473124+(previous day’s mean daily air temperature*0.576203)”.

### Annual pollen integral (APIn)

Beech’s Annual Pollen Integral (APIn) data from 1976 to 2022 (Supplementary Fig. 1) were provided by the Medical University of Vienna. Pollen measurements were performed with a pollen trap of Hirst design^64^ mounted on a rooftop. From 1976 until 2002, pollen measurements were performed at the Department of Otorhinolaryngology of the University Vienna, Medical University of Vienna (48°13’11’’ N, 16°20’54’’ E, WGS84; height above sea level: 200 m; height above ground level: 20 m). From 2003 until 2022, pollen monitoring continued on the rooftop of the main building of GeoSphere Austria (48°14’56’’ N, 16°21’22’’ E, WGS84; height above sea level: 209 m; height above ground level: 9 m). Pollen data were evaluated as daily airborne pollen concentrations following the minimum recommendations of the Aerobiological community^65^ and the European Standard for the sampling and analysis of airborne pollen grains and fungal spores for networks related to allergy (ÖNORM EN-16868:2019) to ensure data quality. Pollen data were assessed by Siegfried Jäger (†) from 1976 until 2011 and afterwards continued by Maximilian Bastl until 2022. Since airborne pollen abundance is a good predictor of beech fall seed production^37^, we used APIn as a proxy for seed availability. Especially in beech, the abundance of pollen in the air during the pollination period is a dominant factor in ensuring successful fertilization, making the annual pollen density a reliable predictor of masting^66^. Beech mast intensity is, in general, subdivided into mast-failure years (< 30% masting trees), intermediate mast years (30–85% masting trees), and full mast years (> 85% masting trees). Relying only on APIns, it is difficult to differentiate between these three categories^19^. Thus, we classified mast years (> 593 pollen grains/m^3^/year) and mast-failure years (< 593 pollen grains/m^3^/year) for our study by setting the threshold to 593 pollen grains/m^3^/year. This threshold is 30% of the APIn in the year 2001 (with 1,976 pollen grains/m^3^/year), which is the maximum APIn excluding the years 2016 and 2018. The very high pollen densities in the years 2016 and 2018 are showing new dimension of APIns and might be only outliers in the long-term. Thus, using these years as a reference point seems to be not a reliable criterion.

### Statistical analysis

Statistical analyses were performed using the statistic program R Version 4.2.1^67^ and Program MARK Version 9.0^68^. Significance for statistical analyses was accepted at *p* < 0.05 with 95% confidence intervals.

### First capture date of dormice after hibernation related to soil temperature

Linear mixed models (LMMs) were built to examine the variation in first capture date after hibernation. All LMMs were built with “lmer” in the R-package “lme4”^69^, and it was visually confirmed that model assumptions were met^70^. First capture dates are the first capture events of each individual for every year until the end of July. The global model included the continuous variable “soil temperature” (mean soil temperature from October to May), the factor variables “age class”, ”sex”, and ”mast” (whether the year before was a mast or a mast-failure year), and two-way interactions between these variables. “Individual ID” and “year” were used as random factors to account for possible pseudo-replication. The following model selection procedure was carried out for this and subsequent LMMS: Starting from fully parameterized models, model selection was done following the parsimony principle using Akaike’s Information Criterion (AIC)^71^ with “dredge” in the R-package “MuMIn”^72^. We used Likelihood-ratio tests to compare models. If models did not significantly differ, the model with fewer terms was selected^73^.

### Effects of air temperature on beech

To investigate the influence of air temperature on beech APIns, we analysed the mean summer temperature (June + July) of one (temp_year−1_) and two years (temp_year−2_) before the respective year. Mean air temperatures of June and July were included, and August excluded because the weather during flower induction period in June and July before the flowering year has been suggested to have a strong influence on masting^12^. Since the sum mean of temp_year−1_ and temp_year−2_ (= temp_year−1&year−2_) correlated with temp_year−1_ and temp_year−2_, respectively, it was tested separately. However, temp_year−1&year−2_ showed no effect on APIn (F-value = 0.31, df = 15, *p* = 0.5842). Starting from the fully parameterized model (beech APIn ~ temp_year−1_ + temp_year−2_ + temp_year−1_ : temp_year−2_), model selection was done. Furthermore, whether inter-annual variation in APIn differed between the periods was tested with “asymptotic_test2” in the R-package “cvequality”^74^.

### Survival-recapture-probability models

Local survival (ɸ) and recapture probability (*P*) were computed using the R-package “RMark”^75^ and Program MARK^68^. Capture histories were determined based on whether an individual was captured at least once in the respective year, ignoring multiple sightings within one year. Sightings of an individual depend on its survival and its recapture probability. Thus, based on whether an individual was captured, it is possible to estimate survival and recapture probability^68^. We used Cormack-Jolly-Seber (CJS) models to estimate local survival (combined effects of mortality and emigration) and recapture probabilities^76^. Accurate estimation of survival probabilities is based on the ability to distinguish between true mortality and the not-detection of an individual during a sampling event, which is directly linked to the recapture probability^76^. Especially in dormice, which are alive, but less likely to be recaptured during mast-failure years (= years without reproduction) due to estivation^30,40^, it is important to explicitly model the recapture probability to account for this reduced detectability. Otherwise, one would over-estimate the mortality rate of dormice. All CJS models with temperature as a continuous variable failed to numerically converge, so we split the dataset into two periods based on a temperature split point with a strong effect on the average temperature per period. Accordingly, we divided the study period into Period 1 (2006–2013) with an average air temperature of 8.14 °C and Period 2 (2014–2022) with an average air temperature of 8.89 °C. Shifting the split point by one or two years in either direction resulted in weaker temperature differences between the two periods and less significant and non-significant p-values in the t-test.

The global model included “sex” (males, females), “age class” (yearlings, adults), “mast” (mast or mast-failure in the current year), and “period” (Period 1 (2006–2013), Period 2 (2014–2022)). Juveniles (non-reproductive age class) were excluded from this analysis because our focus was on the effect of higher mean air temperature on sexually mature dormice. We attempted to fit a global model with all interactions of survival probability ɸ(sex*age class*mast*period) and recapture probability *P*(sex*age class*mast*period). Due to a lack of model fit as a consequence of an excessive number of estimated parameters, we selected a model with fewer interactions (maximum three-way interactions). All variations of the global model of ɸ and *P* are shown in Supplementary Table 2. Goodness-of-fit (GOF) was tested with “release.gof” in the R-package “RMark” (χ² = 132.85, df = 145, *p* = 0.7565). The median-c-hat approach implemented in the program MARK was used to test for overdispersion. Analyses of local survival and recapture probabilities were corrected with a c-hat of 1.2528075 to count for overdispersion. As the last estimate for survival and recapture probability cannot be separated at CJS models, we created a distinct factor level for the last occasions, which is not included in the results shown. Models considered at model selection were built by combining all variables for ɸ and *P*, including both additive effects and interactions. Model selection was done based on the quasi-likelihood corrected Akaike information criterion (QAICc)^77^ and followed the parsimony principle. Firstly, we fixed ɸ to the global models shown in Table S1 and ran all combinations for P. Afterwards, we fixed P to the global models shown in Table S1 and the best models of the first step with a QAICc smaller than 2 and ran all combinations for ɸ. The model with the lowest QAICc score was chosen as the best model, and all models with a ΔQAICc score smaller than 2 were considered in the results. The results show the estimates and 95% confidence intervals of the indicated model’s survival and recapture probabilities. Non-overlapping confidence intervals represent significant differences.

### Reproductive activity of females, litter size, and body mass of dormice

LMMs were built to examine the variation in litter size. Furthermore, general linear mixed models (GLMMs; build with “glmer” in the R-package “lme4”) with a binominal error distribution and logit-link function examined the variation in the proportion of reproductively active females. The global model included the continuous variable “beech APIn” (of the current year), the factor variables “age class” and “period”, and two-way interactions between these variables. In the GLMM explaining the variation in the proportion of reproductively active females, the interaction between “age class” and “period” was not possible due to the low number of reproductively active yearlings in Period 2. “Individual ID” and “year” were used as random factors to account for possible pseudo-replication. Model selection of GLMMs was done using the same procedure as for LMMs (see section “First capture date of dormice after hibernation related to soil temperature”). The random factor “Individual ID” was removed from the final models since it had no effect on the response variable after accounting for the fixed effects.

Furthermore, we compared body mass of yearlings and adults at the start of the active season over the two periods using a Type III ANOVA. We then used “glht” in the R-package “multcomp”^78^ to assess specific group differences. The three-way interaction of period, age class, and whether the year before hibernation was a mast or mast-failure year was not significant (F-value_1,517_ = 0.10, *p* = 0.7508).

## Supplementary Information

Below is the link to the electronic supplementary material.

Supplementary Material 1

## Data Availability

The datasets analysed during the current study are available at Dryad Digital Repository (after acceptance of the manuscript).
