# Supplementary material for: Indirect effects of higher mean air temperature related to climate change on major life-history traits in a pulsed-resource consumer
Source: Sci Rep. 2026 Jan 23;16:6050. doi: 10.1038/s41598-026-37071-3 (PMC12902009; doi:10.1038/s41598-026-37071-3)
Supplement: Supplementary file 1 — Supplementary Material 1 [file 41598_2026_37071_MOESM1_ESM.docx]

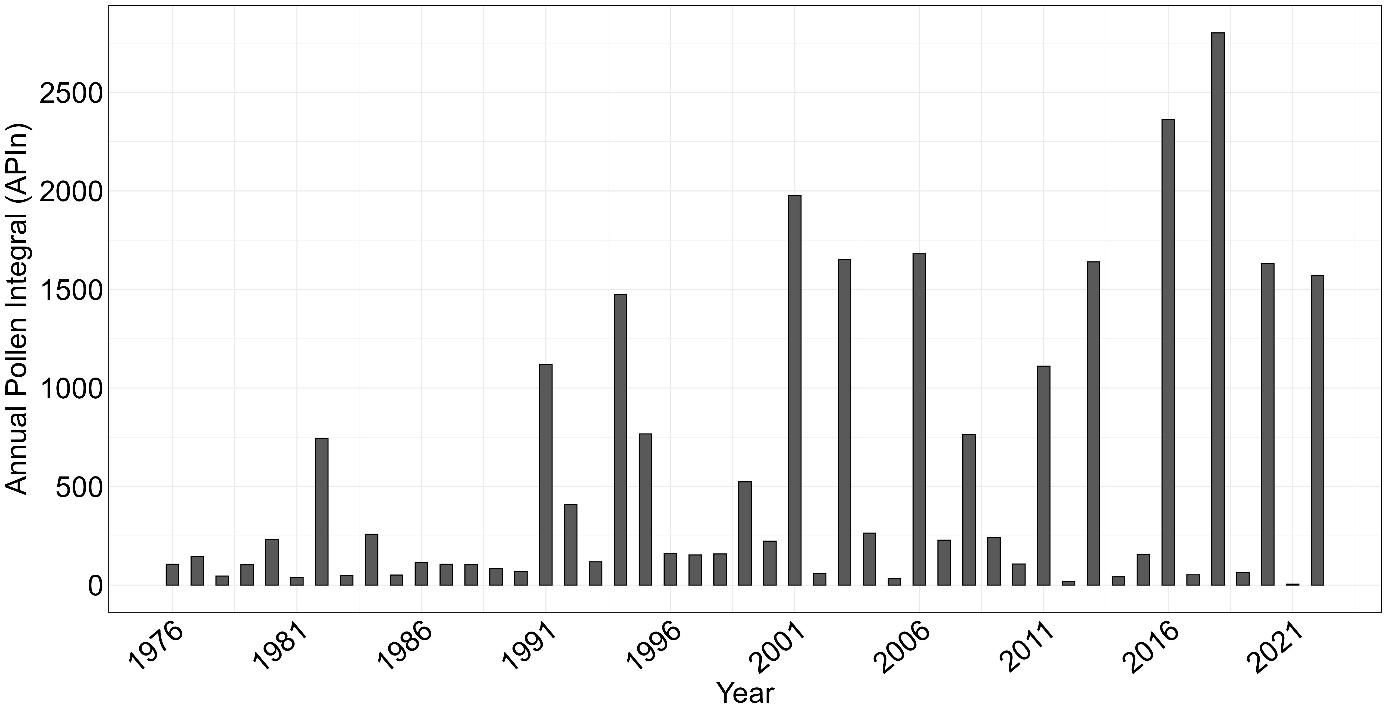
 **Supplementary Figure 1: Annual Pollen Integral (APIn) of the European beech (*Fagus sylvatica*) from 1976 to 2022.** Data were provided by the Medical University of Vienna.


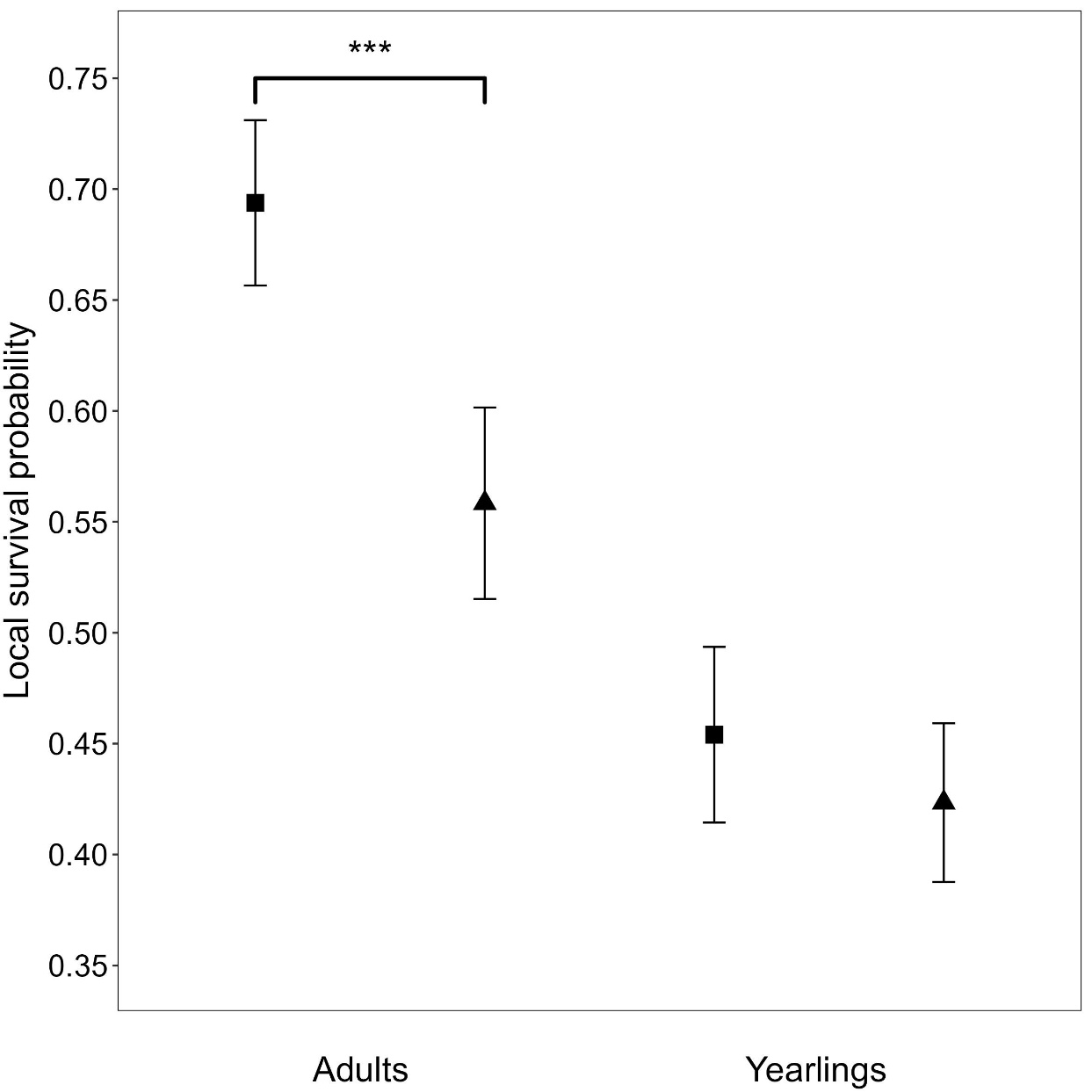

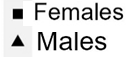

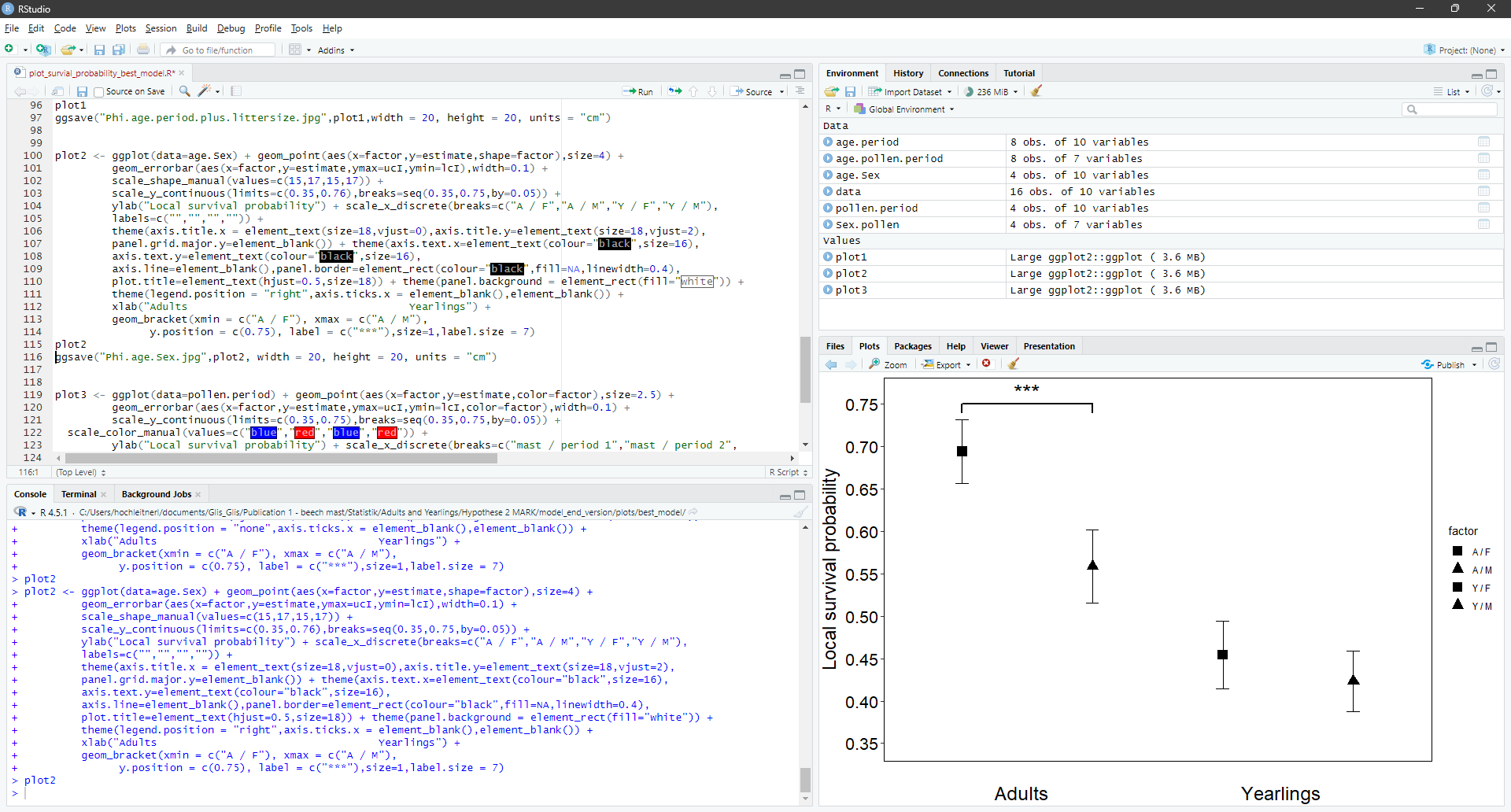


**Supplementary Figure 2: Local annual survival probability (mean estimate with 95% confidence intervals) of dormice (n = 2,530).** Local annual survival probability of adults and yearlings differentiated per sex from the best capture-recapture model. Stars and brackets indicate statistically significant differences, but no absolute p-value can be determined when using MARK.


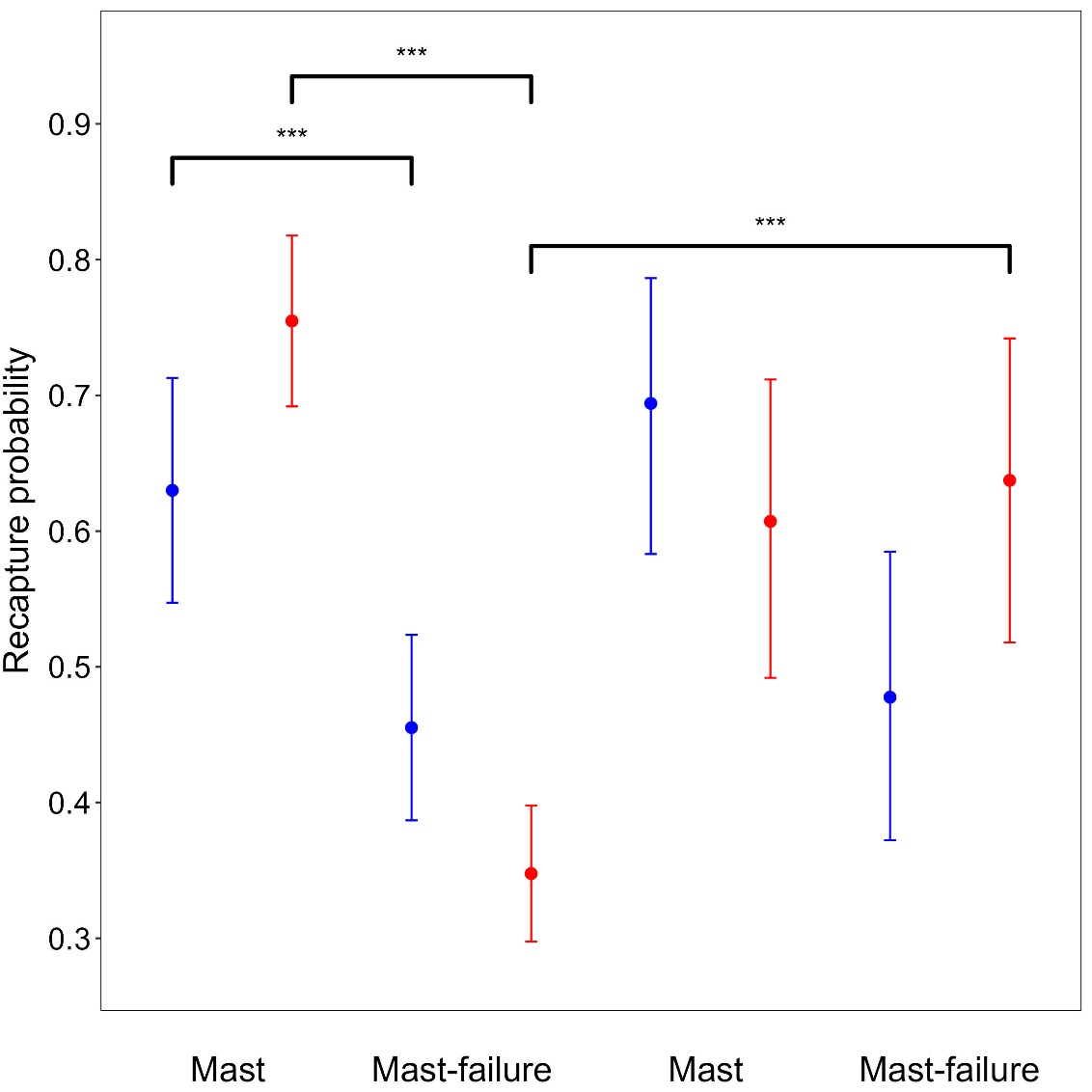

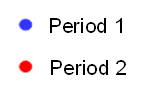


**Supplementary Figure 3: Recapture probability (mean estimate with 95% confidence intervals) of dormice (n = 2,530).** Recapture probability of adults and yearlings during mast years and mast-failure years over period 1 and period 2 from the best capture-recapture model. Stars and brackets indicate statistically significant differences, but no absolute p-value can be determined when using MARK.

**Supplementary Figure 4: Correlation of daily mean air temperatures.** (A) Observed strong correlation between daily mean air temperatures recorded at the study area and the weather station in Berndorf (Pearson’s correlation: rho = 0.97). Each data point (5441 in total) represents a daily mean air temperature. (B) Daily mean air temperature (°C) from January 1^st^, 2006, to December 31^st^, 2022, for both the study area (shown as dark-red solid line) and the weather station in Berndorf (shown as stell-blue dashed line). During periods of missing data from the study area (in total 768 days due to measurement errors), only the temperature from the weather station in Berndorf is displayed.

**A**


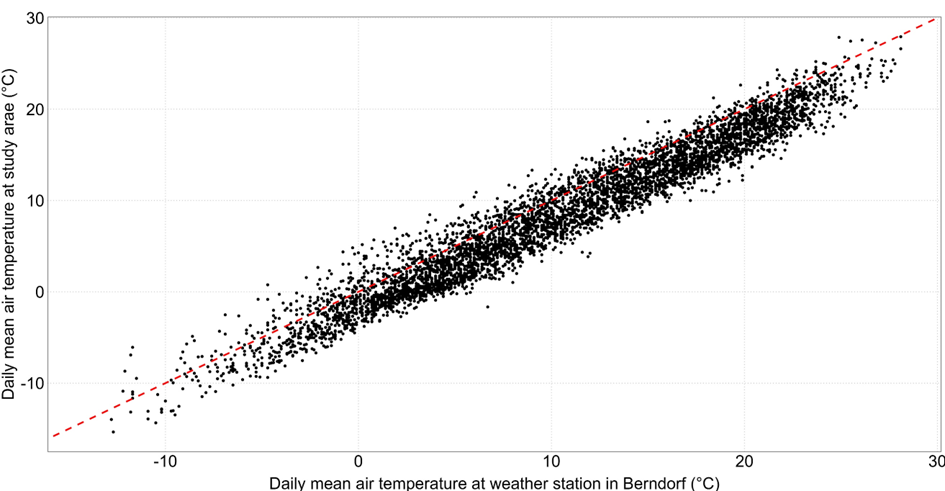

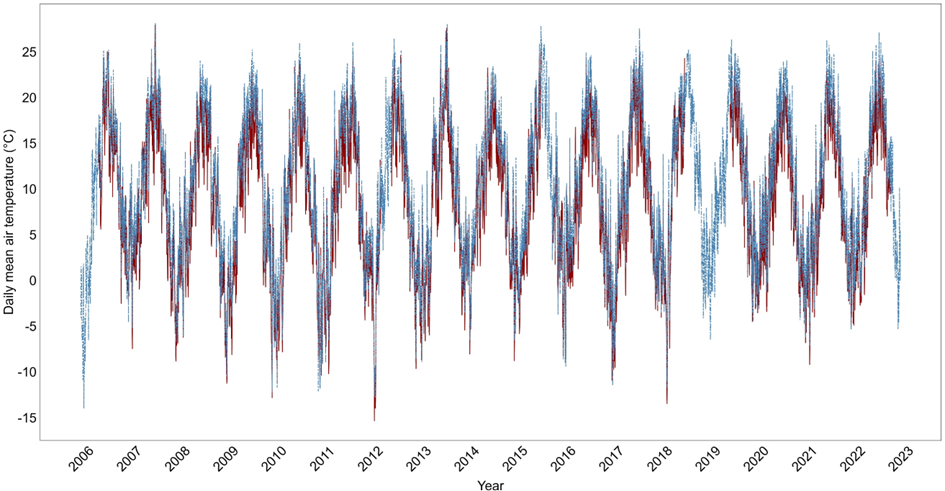


**B**

**Supplementary Table 1: Results of linear mixed-effects model explaining direct effects of mean soil temperature on dormice.** Mean soil temperature (i.e., hibernacula) form October to May had no signfiicant influence on first capture dates after hibernation (t-value_1,2939_ = 0.17, χ² = 0.39, p = 0.5307). Final linear mixed effect model explaining the variation in the first capture date after hibernation (n = 2946 (number of observations), N = 2057 (number of individuals)). Reference levels of factor variables: sex_female_, age class_adult_, previous year_mast failure_. R²m = 0.18, R²c = 0.31.

| (A) | **estimate** | **SE** | **χ²** | **p-value** |
| --- | --- | --- | --- | --- |
| Intercept | 178.78 | 9.33 | 367.13 | < 0.0001 |
| Sex_male_ | -12.82 | 0.88 | 254.97 | < 0.0001 |
| Age class_yearling_ | -14.88 | 8.40 | 13.49 | 0.0002 |
| Previous year_mast_ | 9.69 | 1.68 | 39.34 | < 0.0001 |
| Soil temperature | 0.26 | 1.48 | 0.39 | 0.5307 |
| Sex_male_ : Age class_yearling_ | 3.89 | 1.43 | 7.42 | 0.0065 |
| Age class_yearling_ : Previous year_mast_ | 3.90 | 2.45 | 2.53 | 0.1116 |
| Age class_yearling_ : Soil temperature | 2.01 | 1.45 | 1.92 | 0.1664 |

**Supplementary Table 2**: **List of used global models of survival ɸ and recapture probability *P*, respectively, with in total a maximum of 22 parameters**. The models include the following variables: ”sex” (males and females), “age class” (yearlings and adults), “mast” (mast years and mast-failure years) and “period” (Period 1 (2006-2013) and Period 2 (2014-2022)). The models include additive effects (“+”) and interactions (“*”).

| age class * sex * mast + period * sex |
| --- |
| age class * sex * mast + period * age class |
| age class * sex * period + sex * mast |
| age class * sex * period + age class * mast |
| age class * mast * period + sex * age class |
| age class * mast * period + sex * mast |
| sex * mast * period + age class * mast |
| sex * mast * period + age class * period |
